# Spatiotemporal trends in the discovery of new swine infectious agents

**DOI:** 10.1186/s13567-015-0226-8

**Published:** 2015-09-28

**Authors:** Guillaume Fournié, Lianne Kearsley-Fleet, Joachim Otte, Dirk Udo Pfeiffer

**Affiliations:** Veterinary Epidemiology, Economics and Public Health Group, Department of Production and Population Health, Royal Veterinary College, University of London, London, UK; Food and Agriculture Organization of the United Nations, Regional Office for Asia and the Pacific, Bangkok, Thailand

## Abstract

A literature review was conducted to assess the spatiotemporal trend and diversity of infectious agents that were newly found in pigs between 1985 and 2010. We identified 173 new variants from 91 species, of which 73 species had not been previously described in pigs. These new species, of which one third was zoonotic, were taxonomically diverse. They were identified throughout the study period, predominantly in the main pork producing countries, with the rate of discovery of new virus variants doubling within the last 10 years of the study period. Whilst infectious agent species newly detected in high-income countries were more likely to be associated with higher virulence, zoonotic agents prevailed in low- and middle-income countries. Although this trend is influenced by factors conditioning infectious agent detection – diagnostic methods, surveillance efforts, research interests –, it may suggest that different scales and types of production systems promote emergence of certain types of infectious agents. Considering the rapid transformation of the swine industry, concerted efforts are needed for improving our understanding of the factors influencing the emergence of infectious agents. This information then needs to inform the design of risk-based surveillance systems and strategies directly mitigating the risk associated with these factors.

**Electronic supplementary material:**

The online version of this article (doi:10.1186/s13567-015-0226-8) contains supplementary material, which is available to authorized users.

## Table of contents

1. Introduction

2. Materials and methods

3. Results

1.1 Taxonomic diversity

1.2 Temporal patterns

1.3 Spatial patterns

1.4 Host range

1.5 Context of discovery

4. Discussion

5. Conclusions

6. Competing interests

7. Authors’ contributions

8. Acknowledgements

9. References

## 1. Introduction

**Figure 1:**
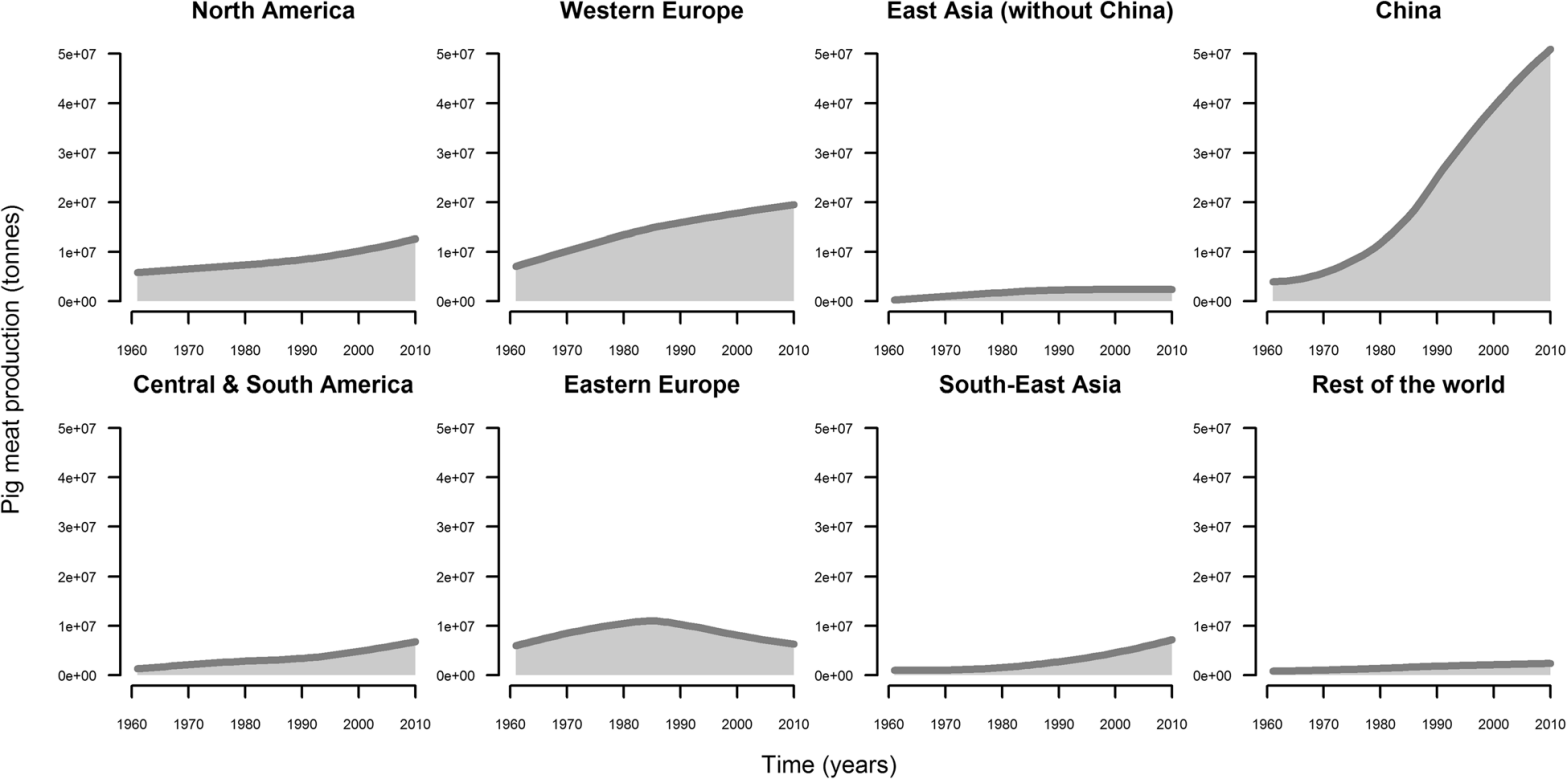
**Evolution of pig meat production as a function of time.** Annual pig production is expressed in tonnes for each country, or group of countries [1].

To meet an increasing demand for meat and meat products, the global pig production sector has experienced rapid growth over the last decades. From 1985 to 2010, global pork production has increased by 80% and become the main meat production sector [1]. The expansion was particularly marked in China (Figure 1) which now accounts for around 50% of the global pig production [1]. This rapid growth was associated with an intensification of production and major transformation of associated value chains. Intensive farms, where large numbers of pigs are kept at high density and raised with a high population turn-over, are often located in areas with high pig farm and pig density [2]. It has been suggested that such high geographical concentration and housing density of domestic animals may allow pathogens to be amplified and to spread rapidly between herds (or flocks) [3-5], resulting in large outbreaks, the mitigation of which requires costly interventions. Novel infectious agents of pigs arising from or being introduced into such areas may therefore cause substantial economic losses, as well as jeopardize food security in affected countries. Porcine reproductive and respiratory syndrome (PRRS) and post-weaning multisystemic syndrome (PMWS) are examples of newly emerged infectious agents which are amongst the swine diseases associated with the highest economic losses since 1985 [6,7].

Novel infectious agents of pigs can also represent a potential threat for public health. Indeed, most emerging human pathogens are of zoonotic origin [8-10], and pigs are a known reservoir for some of them (e.g. *Streptococcus suis*). Moreover, influenza A viruses circulating in pigs may be involved in the generation of novel pandemic strains [11,12]. Pig populations may also act as intermediate hosts, amplifying infectious agents transmitted from other wild or domestic animal species, and then transmitting them to humans (e.g. Nipah virus). The development of pig farming in peri-urban environments enhances the proximity of pig production units to areas of high human population density, creating an interface conducive to the transmission of pathogens, to which humans may otherwise not be exposed. The study of the spatiotemporal trend of swine infectious agent discovery is, therefore, of importance for pig health and welfare, as well as public health.

A literature review was conducted in order to assess the diversity of infectious agents that have been newly found to infect domestic swine (*Sus scrofa domesticus*) under natural transmission conditions between 1985 and 2010, and the spatiotemporal pattern of these discoveries. A new infectious agent was either an infectious agent species not previously found to infect domestic swine, or a new variant of an infectious agent species already known to infect domestic swine. In the latter case, the new variant was either a new serotype, or exhibited distinctive virulence features. Infectious agents are described according to their taxonomy, their host range, the date, location and context of detection.

## 2. Materials and methods

New infectious agents were identified through a literature review. Searches were conducted on PubMed, ISI Web of Knowledge and CAB Direct databases (publication dates between January 1985 and February 2012), using the Boolean search criteria: B1 AND B2 AND B3, with:

- B1: “swine” OR “porcine” OR “pig*” (in the abstract or in the title),
- B2: “pathogen*” OR ‘virus*’ OR “bacteria” OR “fung*” OR “parasit*” OR “helminth*” OR “protozoa*” OR “infection*” OR “disease*” (in the abstract or in the title), and
- B3: “outbreak*” OR “epidemic*” OR “emerg*” OR “case*” (in the abstract or in the title).

Only articles in English were considered. To be included, infectious agents had to be detected from domestic pigs infected under natural conditions through virus isolation or molecular methods, such as polymerase chain reaction (PCR) or sequencing. If only serological evidence was available, swine susceptibility to the infectious agent had to be demonstrated through experimental infection. The date and location of a discovery were defined as the date and location of the sampling of the domestic pig that led to the first identification of the infectious agent. If this information was not provided in the publication or in GenBank [13], the date of article submission or publication and the country of the first author were used instead. For some infectious agents, serological (e.g. hepatitis E virus) or clinical (e.g. porcine reproductive and respiratory syndrome virus) evidence of their occurrence in pigs existed prior to their actual detection. The date and location of this first serological or clinical evidence was then used as the date and location of discovery.

The following micro- and macro-parasite groups were considered: viruses, bacteria, fungi, protozoa and helminths. In the following, they will all be referred as infectious agents. Taxonomic classification was based on the National Centre for Biotechnology Information [14] for all infectious agent types, as well as the International Committee on the Taxonomy of Viruses [15] for viruses, and textbooks for bacteria, fungi [16,17], helminths and protozoa [17,18]. For some infectious agents that have been recently identified, species names have not yet been validated by the relevant Committees responsible for approving the taxonomic classification. The nomenclature proposed in the scientific literature for naming these new infectious agents was then used.

Newly discovered infectious agent variants and species were classified according to the activity that led to their identification, which included (i) “outbreak investigation”, and (ii) “screening or research activities”. The former category included infectious agents that were likely to have been responsible for the investigated outbreak, while the latter category included infectious agents identified during outbreak investigations, but which were unlikely to have caused the outbreak under investigation. Infectious agent species were also characterized by their zoonotic potential. The list of the new swine infectious agents and their characteristics is presented in Additional file 1.

Statistical data on pig meat production was available from the Food and Agriculture Organization of the United Nations (2012). The level of pig meat (pork) production of a given country was defined as its average pig meat production reported in [1] between 1985 and 2010. The World Bank classification was used to differentiate between high-income countries (HIC), and low- and medium-income countries (LMIC) [19]. The strength of association between infectious agent host range or circumstances of discovery and the location of their discovery was assessed through calculation of risk ratios (RRs). This was the ratio between the proportions of infectious agent discoveries with a given feature *k* in two mutually-exclusive country groups, expressed as: $\left({n}_{k,A}/{\displaystyle \sum_i{n}_{i,A}}\right)/\left({n}_{k,B}/{\displaystyle \sum_i{n}_{i,B}}\right)$, where *n*_*k*,*A*_ is the number of discoveries with a feature *k*, e.g. being swine-specific, reported in country group A, e.g. HIC. Confidence intervals (CIs) were estimated by non-parametric bootstrapping [20]. In short, simulated values of *n*_*k*,*A*_ and *n*_*k*,*B*_ were generrated through two binomial processes $B\left({\displaystyle \sum_i{n}_{i,A}},{n}_{k,A}/{\displaystyle \sum_i{n}_{i,A}}\right)$ and $B\left({\displaystyle \sum_i{n}_{i,B}},{n}_{k,B}/{\displaystyle \sum_i{n}_{i,B}}\right)$, respectively, and a simulated RR was computed. This algorithm was repeated 10 000 times, and the 2.5% and 97.5% quantiles of the resulting distribution of the simulated RRs were the lower and upper bounds of the 95% CI. Statistical association between a feature *k* and the location of the discovery was further assessed through a Fisher’s exact test. All analyses were run using R 3.0.1 [21].

## 3. Results

### 3.1 Taxonomic diversity

A total of 173 infectious agent variants newly found to infect domestic swine between 1985 and 2010 were identified through a literature review. Their characteristics and taxonomic diversity are described for each infectious agent type in Table 1. Infectious agent species to which these new variants belonged are further described in Additional files 2 and 3. Almost all of them were either bacteria (54%) or viruses (43%) – mainly RNA viruses (77% of these viral variants). Fungi, helminths or protozoa only accounted for 3% of these new variants. The lower average number of variants per taxon for viruses, and especially DNA viruses, compared to bacteria suggested that newly discovered viruses were more diverse taxonomically (Table 1).

**Table 1:** **New infectious agent variants and species, their characteristics and taxonomic diversity**

	All types	Virus (All)	Virus (DNA)	Virus (RNA)	Bacteria	Others
New infectious agent variants						
.New variants (%)	173 (100%)	74 (43%)	17 (10%)	57 (33%)	93 (54%)	6 (3%)
.Taxonomic diversity						
..Species inc. new variants (*d*)	91 (1.9)	42 (1.8)	17 (1)	25 (2.3)	43 (2.2)	6 (1)
..Genera inc. new variants (*d*)	63 (2.7)	32 (2.3)	10 (1.7)	22 (2.6)	26 (3.6)	5 (1.2)
..Families inc. new variants (*d*)	47 (3.7)	21 (3.5)	6 (2.8)	15 (3.8)	21 (4.4)	5 (1.2)
.Country group						
..HIC (%)	138 (80%)	44 (59%)	12 (71%)	32 (56%)	91 (98%)	3 (50%)
..LMIC (%)	35 (20%)	30 (41%)	5 (29%)	25 (44%)	2 (2%)	3 (50%)
.Host Range						
..From swine-sp. species (%)^a^	64 (37%)	39 (53%)	17 (100%)	22 (39%)	25 (27%)	0 (0%)
…In HIC (%)	51 (37%)	26 (59%)	12 (100%)	14 (44%)	25 (27%)	0 (0%)
…In LMIC (%)	13 (37%)	13 (43%)	5 (100%)	8 (32%)	0 (0%)	0 (0%)
…RR (CI, p)	1 (0.6-1.8, *p* = 1)	1.4 (0.9-2.4, *p* = 0.2)	-	-	-	-
..From zoonotic species (%)^a^	95 (55%)	33 (45%)	0 (0%)	33 (58%)	58 (62%)	4 (67%)
…In HIC (%)	75 (54%)	17 (39%)	0 (0%)	17 (53%)	56 (62%)	2 (67%)
…In LMIC (%)	20 (57%)	16 (53%)	0 (0%)	16 (64%)	2 (100%)	2 (67%)
…RR (CI, p)	1 (0.7-1.4, *p* = 0.9)	0.7 (0.4-1.2, *p* = 0.2)	-	-	-	-
.Context of detection						
..Outbreak investigation (n,%)	58 (144, 40%)	18 (68, 26%)	2 (17, 12%)	16 (51, 31%)	39 (70, 56%)	1 (6, 17%)
…In HIC (%)	51 (112, 46%)	12 (41, 29%)	2 (12, 17%)	10 (29, 34%)	38 (68, 56%)	1 (3, 33%)
…In LMIC (%)	7 (32, 22%)	6 (27, 22%)	0 (5, 0%)	6 (22, 27%)	1 (2, 50%)	0 (3, 0%)
…RR (CI, p)	2.1 (1.2-5.4, *p* = 0.02)	1.3 (0.6-4, *p* = 0.6)	-	-	-	-
New infectious agent species						
.New species (%)	73 (100%)	35 (48%)	17 (23%)	18 (25%)	32 (44%)	6 (8%)
..Unknown before disc. in pigs	50 (68%)	27 (37%)	17 (23%)	10 (14%)	22 (30%)	1 (1%)
..Known to infect other hosts	23 (32%)	8 (11%)	0 (0%)	8 (11%)	10 (14%)	5 (7%)
.Taxonomic diversity						
..Genera inc. new species (*d*)	52 (1.4)	26 (1.3)	10 (1.7)	16 (1.1)	21 (1.5)	5 (1.2)
..Families inc. new species (*d*)	44 (1.7)	19 (1.8)	6 (2.8)	13 (1.4)	20 (1.6)	5 (1.2)
.Country group						
..HIC (%)	54 (74%)	20 (57%)	12 (71%)	8 (44%)	31 (97%)	3 (50%)
..LMIC (%)	19 (26%)	15 (43%)	5 (29%)	10 (56%)	1 (3%)	3 (50%)
.Host Range						
..From swine-sp. species (%)	37 (51%)	24 (69%)	17 (100%)	7 (39%)	13 (41%)	0 (0%)
…In HIC (%)	30 (56%)	17 (85%)	12 (100)	5 (62%)	13 (42%)	0 (0%)
…In LMIC (%)	7 (37%)	7 (47%)	5 (100%)	2 (20%)	0 (0%)	0 (0%)
…RR (CI, p)	1.5 (0.9-3.5, *p* = 0.2)	1.8 (1.1-4, *p* = 0.03)	-	-	-	-
..From zoonotic species (%)	24 (33%)	9 (26%)	0 (0%)	9 (50%)	11 (34%)	4 (67%)
…In HIC (%)	14 (26%)	2 (10%)	0 (0%)	2 (25%)	10 (32%)	2 (67%)
…In LMIC (%)	10 (53%)	7 (47%)	0 (0%)	7 (70%)	1 (100%)	2 (67%)
…RR (CI, p)	0.5 (0.3-1, *p* < 0.05)	0.2 (0–0.7, *p* = 0.02)	-	-	-	-
.Context of detection						
..Outbreak investigation (n,%)	17 (23%)	6 (17%)	2 (12%)	4 (22%)	10 (31%)	1 (17%)
…In HIC (%)	16 (30%)	5 (25%)	2 (17%)	3 (38%)	10 (32%)	1 (33%)
…In LMIC (%)	1 (5%)	1 (7%)	0 (0%)	1 (10%)	0 (0%)	0 (0%)
…RR (CI, p)	5.6 (1.5-∞, *p* = 0.03)	3.8 (0.8-∞, *p* = 0.2)	-	-	-	-

“Others” refer to fungi, helminths and protozoa; d: average number of new variants (or new species) per taxon; HIC high-income countries; LMIC low- and middle-income countries; RR risk ratio; p: Fisher’s exact test p-value. RRs were only computed for all data and viruses, because of the low number of variants and species in one or the two country groups for other infectious agent types.^**a**^ The host range of the species to which new variants belonged was taken into account.

The distribution of new variants as a function of infectious agent species was right-skewed, meaning that most new variants were accounted by only a small number of species. Two (5%) out of the 42 virus species for which new virus variants were reported, Influenza A virus and Rotavirus A, and 2 (5%) out of the 43 bacteria species for which new bacteria variants were reported, *Streptococcus suis* and *Enterococcus faecalis*, accounted for more than a third of all new variants within each infectious agent type. The distribution of new variants per species also depended on the proportion of the study period for which these species had been known to infect swine. Of the 91 infectious agent species that included new variants, 73 species had not been described in swine before 1985 (referred as new species). All but 3 of these new species consisted of only one variant, whereas species known to infect swine before 1985 had an average number of 5.3 new variants (2.6 when discarding the four abovementioned species accounting for most new variants).

Of the 73 new species, 50 species were unknown prior to their detection in swine, while 23 species were known to infect other host species prior to their detection in swine. The low average number of new species per genus or family meant that newly discovered infectious agent species were highly diverse taxonomically (Table 1).

### 3.2 Temporal patterns

**Figure 2:**
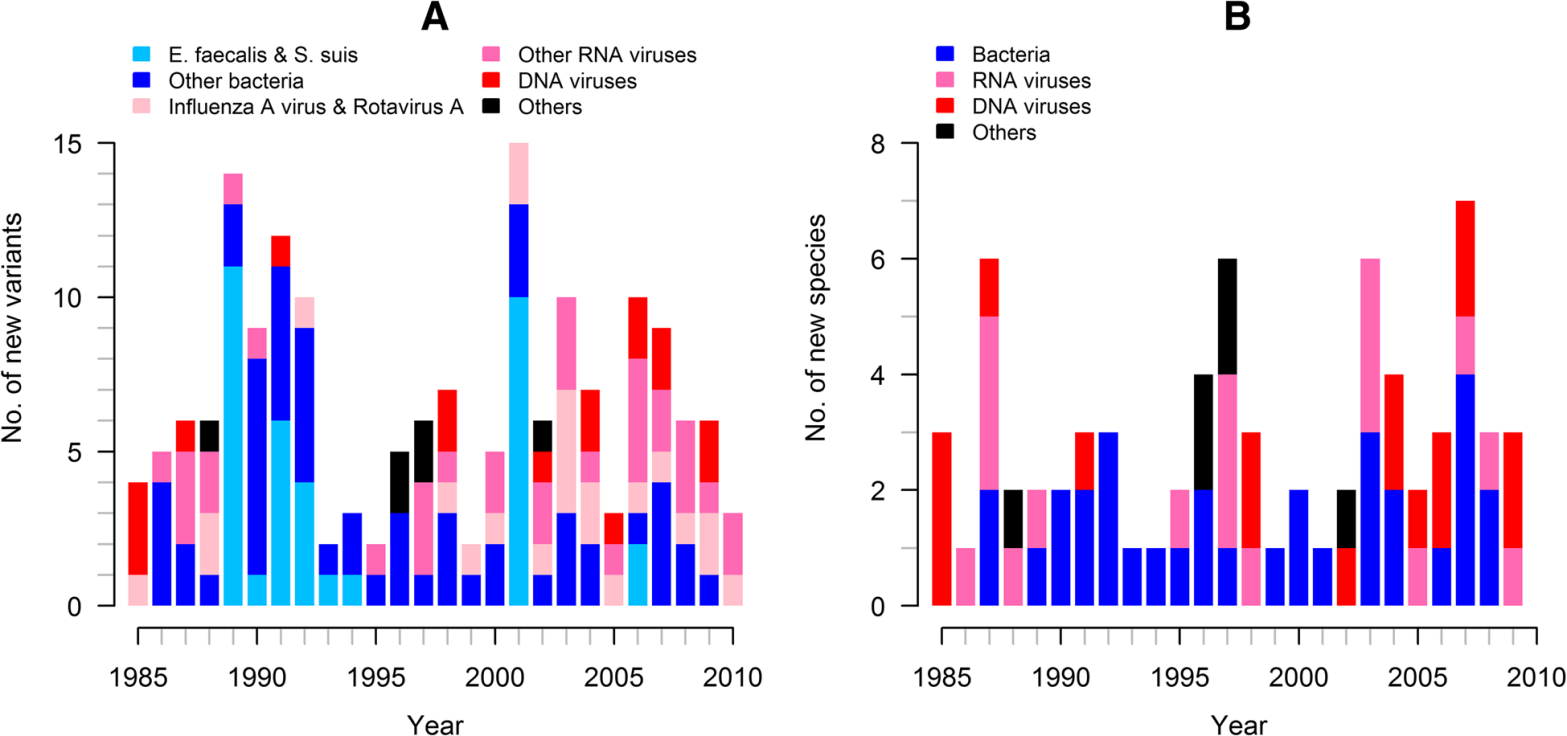
**Temporal trend in the identification of new infectious agent variants and species.** The number of newly discovered variants (**A**) and species (**B**) is shown for each year of the study period.

Figure 2 shows the number of infectious agent variants and species newly found to infect domestic swine as a function of time. Novel variants and species were identified at an average annual rate of 6.7 (range: 2–15) and 2.8 (0–7) over the study period, respectively.

There was no dramatic change in the temporal pattern of identification of new infectious agent variants, with only a slight increase in the annual discovery rate in the past 10 years, from 6.1 between 1985 and 2000 to 7.5 between 2001 and 2010. However, the temporal pattern of discoveries of virus and bacteria differed. While the annual rate of discovery of new bacterial variants decreased from 4 before 2000 to 2.9 after 2000, this rate more than doubled for viral variants over the same period, from 1.8 before 2000 to 4.5 after 2000.

### 3.3 Spatial patterns

**Figure 3:**
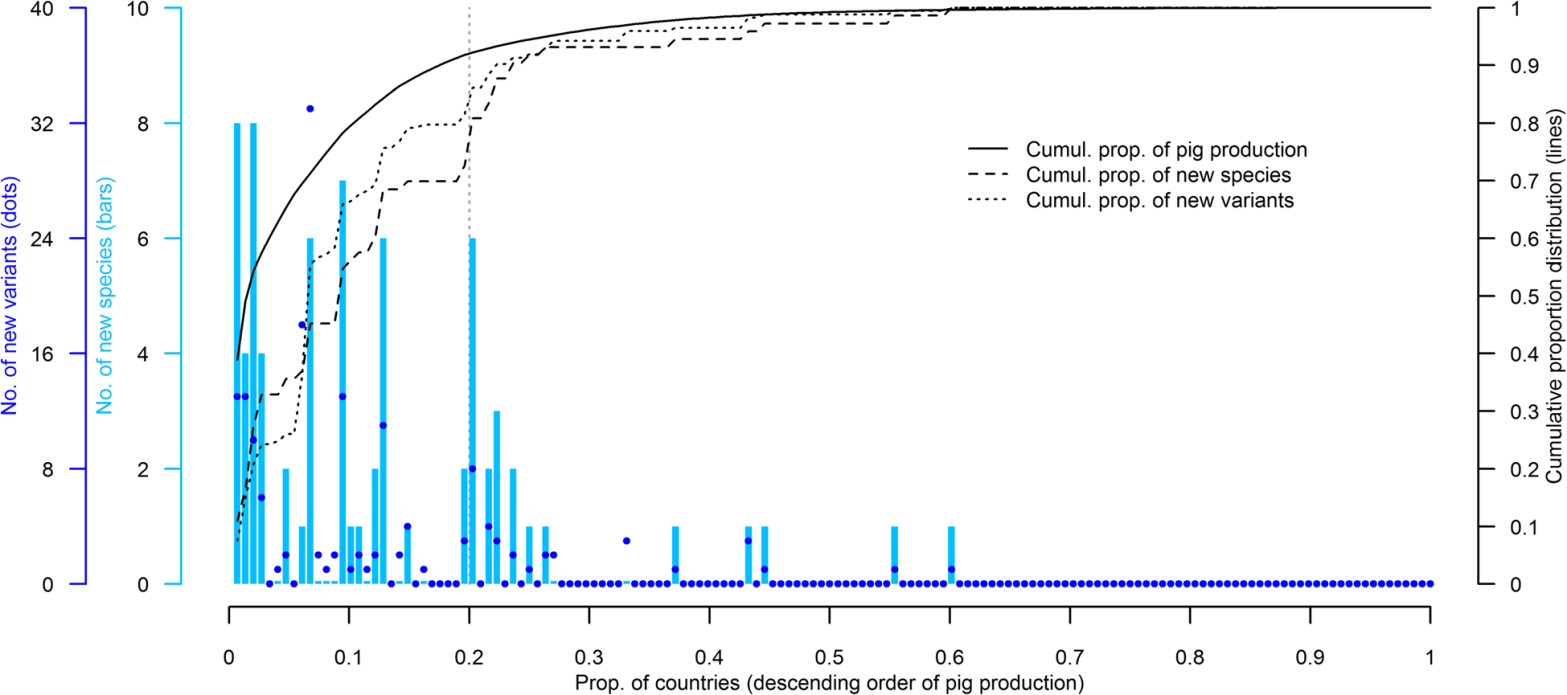
**Number of novel infectious agent variants and species by country.** The number of novel variants (dots) and species (bars) is shown for each country. Countries are arranged in descending order of pig production [1]. The cumulative distributions of new infectious agent variants (dotted line), species (dashed line) and of pig production (solid line) are presented.

Western Europe, North America, Australia and East and South-East Asia accounted for 87% and 86% of all new infectious agent variants and species, respectively. New variants originated in 34 countries and new species in 25, with 58% and 62% of these variants and species having originated in only 7 countries, namely Australia, Canada, China, Germany, Japan, the United Kingdom, and the United States of America (Additional file 4). Countries where new species were reported were also the largest pig meat producers. The top 20% of pig meat-producing countries (based on average pig meat production between 1985 and 2010 [1]) accounted for 92% of global pig meat production, and for 86% and 81% of novel infectious agent variants and species over the study period (Figure 3). Of the 50 largest pig meat producing countries in 1985, 17 have increased their production by more than 50%, up to 440%, in 2010 [1]. At least one novel species was discovered in 11 (65%) of these 17 countries over the study period, and in only 9 (27%) of the other 33 countries. Likewise, at least one novel variant was discovered in 14 (82%) of these 17 countries, and in only 15 (45%) of the other 33.

### 3.4 Host range

Swine infectious agents for which the zoonotic potential is unknown or uncertain – such as strains of porcine norovirus, mamastrovirus (astrovirus), sapovirus and picobirnavirus [22-25] – were conservatively considered to be non-zoonotic. Although most (*n* = 57, 63%) swine infectious agent species which included new variants were non-zoonotic, zoonotic species accounted for most new variants (Table 1). A third of the 73 new infectious agent species identified between 1985 and 2010 were zoonotic (Table 1), with this proportion decreasing over time, from 43% before 2000 to 19% afterwards. Of the new infectious agent species, 23 species were known to infect other hosts prior to their detection in swine. Seventy-four percent (*n* = 17) of these species were already known to infect humans, and 26% (*n* = 6) were only known to infect other animal species.

Half of the novel swine infectious agent species (*n* = 37, 51%) identified between 1985 and 2010 were swine-specific, with this proportion increasing over the study period, from 40% before 2000 to 65% afterwards. Whereas all newly discovered DNA viruses were swine-specific, 61% (*n* = 11) of the new RNA viruses could also infect other host species than swine. The new infectious agent species detected in HIC were more frequently swine-specific than those detected in LMIC. Additionally, the new infectious agent species detected in HIC were less frequently zoonotic than those detected in LMIC (Table 1). The numbers involved were small, and only the difference in the zoonotic potential of agents discovered in HIC and LMIC was statistically significant (Table 1). These trends were less pronounced when considering new viral variants, and even absent when considering all new variants (Table 1). For this analysis of variants, the host range was considered to be not the host range of the variant itself, but rather the host range of the species to which the variants belonged.

### 3.5 Context of discovery

The proportion of new variants and species detected as a result of outbreak investigations was higher for bacteria than viruses (Table 1). The detection of other variants was either fortuitous – infectious agents were identified during an outbreak investigation but were unlikely to have caused the outbreak –, occurred during infectious agent screening, or was motivated by research purposes. Novel infectious agent species reported in HIC were more frequently discovered during outbreak investigations than during infectious agent screening or research activities, when compared with LMIC. A similar but less pronounced trend was observed for new variants (Table 1).

## 4. Discussion

New infectious agents included (i) infectious agents that had been circulating in pigs for extended periods but remained undetected until recently, and (ii) infectious agents that had newly emerged in pigs via a host species jump or mutation. The observed spatiotemporal pattern is likely to have been influenced by advances in diagnostic methods and variations in surveillance efforts. This includes the development of broad-range PCR and meta-genomic analyses [26], reductions in the costs associated with diagnostic testing, spatial and temporal changes in the priorities and sensitivity of active and passive animal health surveillance, and the evolution of research interests [27]. For instance, the discovery of new infectious agent families or genera in a given host species may trigger an active search for related infectious agents in other host species, including pigs (e.g. *Enterococcus faecium* [28], Torque teno virus [29], *Trypanosoma cruzi* [30]). The public health importance of some bacterial and viral species may have motivated a more thorough exploration of their genetic diversity when compared to other infectious agents. This may partly explain the finding that Influenza A viruses, Rotavirus A, *E faecalis* and *S suis* account for more variants than other infectious agent species. The number of new variants was higher in species known to infect swine prior to 1985 than in newly discovered species. This could suggest that the diversity of an infectious agent species depended on the length of time over which it was studied. However, drawing this conclusion would require more information, particularly the number of variants and the time of discovery of all infectious agent species known to infect swine, including those for which no new variants were discovered after 1985.

Apart from improvements in technical ability and surveillance, intrinsic features of infectious agents may have influenced the observed pattern. The observed broader host range of RNA viruses compared to DNA viruses may be due to their higher nucleotide substitution rate during replication. The resulting higher genetic diversity is likely to increase the likelihood of successful host species jumps [31].

Moreover, changes in ecological systems in which pigs are raised may promote the emergence of new infectious agents through a host jump or mutation and, therefore, modify the pattern of infectious agent discovery [9,10]. While production systems within HIC and LMIC groups are heterogeneous, the assumption of higher biosecurity standards and more industrial and specialized production units in HIC than in LMIC seems reasonable. In integrated pig production systems, reduced contacts with humans and other animal species may reduce the likelihood of inter-species transmission; however, the high pig density and rate of turn-over may increase the likelihood of intra-species transmission, as evidenced by the spread of PRRS and PMWS [32,33]. The genetic homogeneity of pig populations to some extent may restrict the range of infectious agents with the potential to jump host species [34]. However, when combined with a high population density of pigs, it may in fact provide ideal conditions for a major epidemic in pigs resulting from a successful host species jump (i.e. an increased likelihood that if an agent successfully jumps to pigs, it will spread widely among the pig population) [35]. This may lead to the very rapid spread of infectious agents through regional and even global pig populations as demonstrated by PRRS, PMWS and, more recently, a new variant of porcine epidemic diarrhea virus [36-38]. Even if the pathogen first has limited transmissibility, the aforementioned pig population characteristics may promote infectious agent fitness gain [39-41], leading to the emergence of swine-specific, and more virulent infectious agents [34]. The interface between high density pig populations and potential sources of pathogens (human, other livestock, companion and wild animal populations) becomes particularly permeable for infectious agents when large numbers of pigs are kept at a low level of biosecurity. Such examples include outdoor herds or large numbers of small-scale and backyard farms in rural villages or semi-urban situations. In this scenario the risk of cross-species jumps will be increased, promoting the infection of pigs by agents infecting multiple host species (e.g. Nipah virus [42], *Trichinella papuae* [43]), possibly including humans.

This review is subject to ascertainment bias. Deciding whether a new variant exhibits new virulence features involves a degree of subjectivity. Moreover, the taxonomic classification of some of the newly discovered infectious agents may change in the near future and then modify the results of this analysis. It is also possible that some new infectious agents were missed by the specified search criteria and language limitations. The exclusion of articles written in languages other than English is likely to have resulted in the overestimation of the proportion of discoveries (i) in English-speaking countries, and (ii) towards the end of the study period (if the preference for reporting such discoveries in the scientific literature in English, rather than in other languages, has increased in recent years). As the infectious agents that we reviewed are not necessarily representative of all new infectious agents, our results and especially associations between infectious agent features (host range and context of discovery) and the location of discovery have to be cautiously interpreted. Moreover, reviewing all variants known to infect swine, before and after 1985 would extend the temporal scale of the study, allowing further exploration of the intra-species diversity among variants, and the factors influencing this, e.g. biological factors, time since discovery.

Despite these limitations, the taxonomic diversity and zoonotic potential of newly discovered infectious agents identified in this review support the need for active screening of pig populations. While pig populations may be a major source of human infection for certain infectious agents (e.g. Nipah virus, *Streptococcus suis*), numerous other infectious agents are likely to be mainly transmitted from humans to pigs, rather than from pigs to humans, as observed with some influenza A viruses [44]. However, even if transmission from humans to pigs is the primary direction for cross-species transmission of certain infectious agents, including influenza A viruses, the role played by pig populations in the evolution of these infectious agents, their zoonotic transfer and the emergence of new variants, is likely to remain substantial [45].

Further research is needed to assess the impact of the continued transformation of the pig production system on the emergence of infectious agents. In particular, there is a need to distinguish infectious agents which have recently invaded pig populations from those that have circulated in pigs for extended periods prior to detection, and to better classify countries according to the characteristics of their pig production sector and animal health surveillance systems. Clearly, the recent increase in absolute numbers and concentration of domestic pig populations, particularly in the central and eastern parts of China [46], has been unprecedented, and expansion of pig production still continues. Chinese pork production rose by approximately 300% over the last 30 years [1] and is likely to continue to increase in order to meet the increasing demand for pork [46]. Without doubt the health management of these enormous high-density pig populations will become a tremendous challenge, even if state-of-the-art bio-exclusion and bio-containment methods are used by most holdings. Moreover, recent decades have also seen an increase in the amount of animal feeds, live pigs and pig products being moved throughout the world, with the volume of traded quantities increasing at an even faster rate than that of pig production [1]. Such intensification of trading activities can promote the rapid circulation of infectious agents between pig populations that might have otherwise remained epidemiologically isolated [38,47]. Not only does this create opportunities for jumps to new host species followed by recombination of the infectious agents, it can also facilitate the global spread of the resultant infectious agents.

## 5. Conclusions

The swine infectious agents discovered in recent years are highly diverse, with a substantial proportion being zoonotic. Although the spatiotemporal distribution of these discoveries is influenced by factors which facilitate their detection, it may suggest that different scales and types of production systems promote emergence of certain types of infectious agents. In light of this, global efforts for enhanced risk-based surveillance are required, as well as the development of more effective risk management for emerging infectious agents. This can only be achieved by a better understanding of the mechanisms underlying the emergence of infectious agents, including the role of the ongoing transformation of the global pig production sector.

## Additional files

Additional file 1:
**Characteristics of the new swine infectious agents.** Disc: type of discovery (1: infectious agent species that was unknown before its discovery in pigs, 2: novel variant(s) from an infectious agent species known to infect pigs, 3: first infection of pigs by an infectious agent species known to infect other host species), Cont: context of discovery (1: infectious agent screening or research activities, 2: outbreak investigation), Host range (1: swine-specific, 2: infect other animal species, 3: host range includes humans), *: Only rotavirus variants classified according to the nucleotide-sequence-based classification system, and recognised by the Rotavirus Classification Working Group are considered. The list of references is available upon request to the authors. Additional file 2:
**Infectious agent species for which new variants were identified in swine from 1985 to 2010.** n: number of new variants; Sp: the species is swine-specific; Zoo: the species is zoonotic; Outbreak: number of new variants identified through outbreak investigation; PRRS: Porcine reproductive and respiratory syndrome; PED: Porcine Epidemic Diarrhea; Tt: Torque teno; SHB : Swine hepatitis B; enc.: encephalitis. Additional file 3:
**Infectious agent families for which new species were identified in swine from 1985 to 2010.** n: number of new species; Unknown: number of species which were unknown prior to their detection in swine; Swine-specific: number of new species which are swine-specific; Zoonotic: number of new species which are zoonotic; Outbreak: number of new species identified through outbreak investigation. Additional file 4:
**Infectious agent discoveries per country.** An underlined country name means that this country is among the top 20% pig meat producing countries for the period 1985–2010. The variation in pig meat production from 1985 to 2010 was computed for the countries listed among the 50 largest pig meat producers in 1985. Countries for which this variation was higher than 50% are indicated by an asterisk.
