# Supplementary material for: Spatiotemporal trends in the discovery of new swine infectious agents
Source: Vet Res. 2015 Sep 28;46:114. doi: 10.1186/s13567-015-0226-8 (PMC4584486; doi:10.1186/s13567-015-0226-8)
Supplement: Additional file 2: — Infectious agent species for which new variants were identified in swine from 1985 to 2010. n: number of new variants; Sp: the species is swine-specific; Zoo: the species is zoonotic; Outbreak: number of new variants identified through outbreak investigation; PRRS: Porcine reproductive and respiratory syndrome; PED: Porcine Epidemic Diarrhea; Tt: Torque teno; SHB : Swine hepatitis B; enc.: encephalitis. [file 13567_2015_226_MOESM2_ESM.docx]

| Type | Family | Genus and species | n | Sp | Zoo | Outbreak |
| --- | --- | --- | --- | --- | --- | --- |
| Bacteria | Actinomycetaceae | Actinomyces hyovaginalis | 1 | No | No | 1 (1, 100%) |
| Bacteria | Actinomycetaceae | Actinomyces suimastitidis | 1 | Yes | No | 1 (1, 100%) |
| Bacteria | Actinomycetaceae | Arcanobacterium abortisuis | 1 | Yes | No | 1 (1, 100%) |
| Bacteria | Aeromonadaceae | Aeromonas allosaccharophila | 1 | No | Yes | 0 (1, 0%) |
| Bacteria | Aeromonadaceae | Aeromonas simiae | 1 | No | No | 0 (1, 0%) |
| Bacteria | Brachyspiraceae | Brachyspira hyodysenteriae | 2 | No | No | 2 (2, 100%) |
| Bacteria | Brachyspiraceae | Brachyspira murdochii | 1 | No | No | 0 (1, 0%) |
| Bacteria | Brachyspiraceae | Brachyspira suanatina | 1 | No | No | 1 (1, 100%) |
| Bacteria | Campylobacteraceae | Arcobacter thereius | 1 | No | No | 0 (1, 0%) |
| Bacteria | Campylobacteraceae | Arcobacter trophiarum | 1 | Yes | No | 0 (1, 0%) |
| Bacteria | Campylobacteraceae | Campylobacter hyoilei | 1 | Yes | No | 1 (1, 100%) |
| Bacteria | Campylobacteraceae | Campylobacter hyointestinalis | 1 | No | Yes | - |
| Bacteria | Carnobacteriaceae | Allofustis seminis | 1 | Yes | No | 0 (1, 0%) |
| Bacteria | Clostridiaceae | Clostridium herbivorans | 1 | Yes | No | 0 (1, 0%) |
| Bacteria | Clostridiaceae | Clostridium intestinalis | 1 | No | Yes | 0 (1, 0%) |
| Bacteria | Coriobacterineae | Olsenella umbonata | 1 | No | No | 0 (1, 0%) |
| Bacteria | Cornebacteriaceae | Corynebacterium glucuronolyticum | 1 | No | Yes | 0 (1, 0%) |
| Bacteria | Cornebacteriaceae | Corynebacterium suicordis | 1 | Yes | No | 1 (1, 100%) |
| Bacteria | Corynebacterineae | Corynebacterium ulcerans | 1 | No | Yes | 0 (1, 0%) |
| Bacteria | Enterobacteriaceae | Escherichia coli | 4 | No | Yes | 3 (3, 100%) |
| Bacteria | Enterobacteriaceae | Salmonella enterica | 3 | No | Yes | 0 (3, 0%) |
| Bacteria | Enterococcaceae | Enterococcus faecalis | 12 | No | Yes | - |
| Bacteria | Enterococcaceae | Enterococcus faecium | 1 | No | Yes | 0 (1, 0%) |
| Bacteria | Helicobacteracaeae | Helicobacter suis | 1 | No | Yes | 0 (1, 0%) |
| Bacteria | Lactobacillaceae | Lactobacillus mucosae | 1 | No | Yes | 0 (1, 0%) |
| Bacteria | Leptospiraceae | Leptospira fainei | 1 | No | Yes | 1 (1, 100%) |
| Bacteria | Mycobacteriaceae | Mycobacterium africanum | 1 | No | Yes | 0 (1, 0%) |
| Bacteria | Mycobacteriaceae | Mycobacterium celatum | 1 | No | Yes | 0 (1, 0%) |
| Bacteria | Mycoplasmataceae | Mycoplasma arthritidis | 1 | No | No | 1 (1, 100%) |
| Bacteria | Nocardiaceae | Gordona sputi | 1 | No | Yes | 1 (1, 100%) |
| Bacteria | Pasteurellaceae | Actinobacillus minor | 1 | Yes | No | 0 (1, 0%) |
| Bacteria | Pasteurellaceae | Actinobacillus pleuropneumoniae | 3 | Yes | No | 3 (3, 100%) |
| Bacteria | Pasteurellaceae | Actinobacillus porcinus | 1 | Yes | No | 0 (1, 0%) |
| Bacteria | Pasteurellaceae | Actinobacillus porcitonsillarum | 1 | Yes | No | 0 (1, 0%) |
| Bacteria | Pasteurellaceae | Haemophilus parasuis | 9 | Yes | No | - |
| Bacteria | Pasteurellaceae | Mannheimia haemolytica | 1 | No | Yes | 1 (1, 100%) |
| Bacteria | Pasteurellaceae | Pasteurella multocida | 1 | No | Yes | 1 (1, 100%) |
| Bacteria | Staphylococcaceae | Staphylococcus aureus | 1 | No | Yes | 0 (1, 0%) |
| Bacteria | Staphylococcaceae | Staphylococcus rostri | 1 | Yes | No | 0 (1, 0%) |
| Bacteria | Streptococcaceae | Streptococcus orisratti | 1 | No | No | 1 (1, 100%) |
| Bacteria | Streptococcaceae | Streptococcus orisuis | 1 | Yes | No | 0 (1, 0%) |
| Bacteria | Streptococcaceae | Streptococcus suis | 24 | No | Yes | 19 (24, 79%) |
| Bacteria | Veillonellaceae | Veillonella magna | 1 | Yes | No | 0 (1, 0%) |
| Fungi | Clavicipitaceae | Claviceps Africana | 1 | No | No | 1 (1, 100%) |
| Fungi | Enterocytozoonidae | Enterocytozooan bieneusi | 1 | No | Yes | 0 (1, 0%) |
| Helminths | Trichinellidae | Trichinella papuae | 1 | No | Yes | 0 (1, 0%) |
| Protozoa | Cryptosporididae | Cryptosporidium muris | 1 | No | Yes | 0 (1, 0%) |
| Protozoa | Trypanosomatidae | Trypanosoma cruzi | 1 | No | Yes | 0 (1, 0%) |
| Protozoa | Trypanosomatidae | Trypanosoma vivax | 1 | No | No | 0 (1, 0%) |
| Virus (DNA) | Adenoviridae | Mastadenovirus Porcine adenovirus C | 1 | Yes | No | 1 (1, 100%) |
| Virus (DNA) | Anelloviridae | Iotatorquevirus Tt sus virus 1a | 1 | Yes | No | 0 (1, 0%) |
| Virus (DNA) | Anelloviridae | Iotatorquevirus Tt sus virus 1b | 1 | Yes | No | 0 (1, 0%) |
| Virus (DNA) | Anelloviridae | Kappatorquevirus Tt sus virus k2 | 1 | Yes | No | 0 (1, 0%) |
| Virus (DNA) | Circoviridae | Circovirus Porcine circovirus type 2 | 1 | Yes | No | 1 (1, 100%) |
| Virus (DNA) | Hepadnaviridae | Orthohepadnavirus SHB virus | 1 | Yes | No | 0 (1, 0%) |
| Virus (DNA) | Herpesviridae | Macavirus Suid herpesvirus 3 | 1 | Yes | No | 0 (1, 0%) |
| Virus (DNA) | Herpesviridae | Macavirus Suid herpesvirus 4 | 1 | Yes | No | 0 (1, 0%) |
| Virus (DNA) | Herpesviridae | Macavirus Suid herpesvirus 5 | 1 | Yes | No | 0 (1, 0%) |
| Virus (DNA) | Parvoviridae | Bocavirus Porcine boca-like virus | 1 | Yes | No | 0 (1, 0%) |
| Virus (DNA) | Parvoviridae | Bocavirus Porcine boca-like virus 1 | 1 | Yes | No | 0 (1, 0%) |
| Virus (DNA) | Parvoviridae | Bocavirus Porcine boca-like virus 2 | 1 | Yes | No | 0 (1, 0%) |
| Virus (DNA) | Parvoviridae | Bocavirus Porcine boca-like virus 3 | 1 | Yes | No | 0 (1, 0%) |
| Virus (DNA) | Parvoviridae | Bocavirus Porcine boca-like virus 4 | 1 | Yes | No | 0 (1, 0%) |
| Virus (DNA) | Parvoviridae | Cnvirus Porcine cnvirus | 1 | Yes | No | 0 (1, 0%) |
| Virus (DNA) | Parvoviridae | Hokovirus Porcine hokovirus | 1 | Yes | No | 0 (1, 0%) |
| Virus (DNA) | Parvoviridae | Unassigned 1 Porcine parvovirus 4 | 1 | Yes | No | 0 (1, 0%) |
| Virus (RNA) | Arteriviridae | Arterivirus PRRS virus | 3 | Yes | No | 3 (3, 100%) |
| Virus (RNA) | Astroviridae | Mamastrovirus Porcine astrovirus | 4 | Yes | No | 0 (4, 0%) |
| Virus (RNA) | Bornaviridae | Bornavirus Borna disease virus | 1 | No | No | 0 (1, 0%) |
| Virus (RNA) | Bunyaviridae | Orthobunyavirus Bunyamwera virus | 1 | No | Yes | 0 (1, 0%) |
| Virus (RNA) | Bunyaviridae | Orthobunyavirus California enc. virus | 2 | No | Yes | 0 (2, 0%) |
| Virus (RNA) | Caliciviridae | Norovirus Porcine norovirus | 1 | Yes | No | 0 (1, 0%) |
| Virus (RNA) | Caliciviridae | Sapovirus Porcine sapovirus | 5 | Yes | No | 0 (5, 0%) |
| Virus (RNA) | Caliciviridae | St-Valerien-like virus | 1 | Yes | No | 0 (1, 0%) |
| Virus (RNA) | Coronaviridae | Alphacoronavirus PED Virus | 1 | Yes | No | 1 (1, 100%) |
| Virus (RNA) | Coronaviridae | Betacoronavirus SARS coronavirus | 1 | No | Yes | 0 (1, 0%) |
| Virus (RNA) | Coronaviridae | Torovirus Porcine torovirus | 1 | Yes | No | 0 (1, 0%) |
| Virus (RNA) | Filoviridae | Ebolavirus Reston ebolavirus | 1 | No | Yes | 0 (1, 0%) |
| Virus (RNA) | Flaviviridae | Pestivirus Bungowannah virus | 1 | Yes | No | 1 (1, 100%) |
| Virus (RNA) | Hepeviridae | Hepevirus Hepatitis E virus | 1 | No | Yes | 0 (1, 0%) |
| Virus (RNA) | Orthomyxoviridae | Influenzavirus A Influenza A virus | 12 | No | Yes | 5 (12, 42%) |
| Virus (RNA) | Paramyxoviridae | Henipavirus Nipah virus | 1 | No | Yes | 1 (1, 100%) |
| Virus (RNA) | Paramyxoviridae | Rubulavirus Menangle virus | 1 | No | Yes | 1 (1, 100%) |
| Virus (RNA) | Paramyxoviridae | Rubulavirus Parainfluenzavirus 5 | 1 | No | No | 0 (1, 0%) |
| Virus (RNA) | Paramyxoviridae | Rubulavirus Porcine Rubulavirus | 1 | Yes | No | 1 (1, 100%) |
| Virus (RNA) | Picobirnaviridae | Picobirnavirus Porcine picobirnavirus | 2 | Yes | No | 0 (2, 0%) |
| Virus (RNA) | Picornaviridae | Enterovirus Porcine enterovirus B | 1 | Yes | No | 0 (1, 0%) |
| Virus (RNA) | Picornaviridae | Kobuvirus Porcine kobuvirus | 1 | Yes | No | 0 (1, 0%) |
| Virus (RNA) | Reoviridae | Rotavirus Rotavirus A* | 11 | No | Yes | 3 (5, 60%) |
| Virus (RNA) | Reoviridae | Seadornavirus Banna virus | 1 | No | Yes | 0 (1, 0%) |
| Virus (RNA) | Rhabdoviridae | Vesiculovirus Chandipura virus | 1 | No | Yes | 0 (1, 0%) |
