# Supplementary material for: Spatiotemporal trends in the discovery of new swine infectious agents
Source: Vet Res. 2015 Sep 28;46:114. doi: 10.1186/s13567-015-0226-8 (PMC4584486; doi:10.1186/s13567-015-0226-8)
Supplement: Additional file 3: — Infectious agent families for which new species were identified in swine from 1985 to 2010. n: number of new species; Unknown: number of species which were unknown prior to their detection in swine; Swine-specific: number of new species which are swine-specific; Zoonotic: number of new species which are zoonotic; Outbreak: number of new species identified through outbreak investigation. [file 13567_2015_226_MOESM3_ESM.docx]

| Type | Family | n | Unknown | Swine-specific | Zoonotic | Outbreak |
| --- | --- | --- | --- | --- | --- | --- |
| Bacteria | Actinomycetaceae | 3 | 3 (100%) | 2 (67%) | 0 (0%) | 3 (100%) |
| Bacteria | Aeromonadaceae | 2 | 0 (0%) | 0 (0%) | 1 (50%) | 0 (0%) |
| Bacteria | Brachyspiraceae | 2 | 2 (100%) | 0 (0%) | 0 (0%) | 1 (50%) |
| Bacteria | Campylobacteraceae | 3 | 3 (100%) | 2 (67%) | 0 (0%) | 1 (33%) |
| Bacteria | Carnobacteriaceae | 1 | 1 (100%) | 1 (100%) | 0 (0%) | 0 (0%) |
| Bacteria | Clostridiaceae | 2 | 2 (100%) | 1 (50%) | 1 (50%) | 0 (0%) |
| Bacteria | Coriobacterineae | 1 | 1 (100%) | 0 (0%) | 0 (0%) | 0 (0%) |
| Bacteria | Cornebacteriaceae | 2 | 1 (50%) | 1 (50%) | 1 (50%) | 1 (50%) |
| Bacteria | Corynebacterineae | 1 | 0 (0%) | 0 (0%) | 1 (100%) | 0 (0%) |
| Bacteria | Enterococcaceae | 1 | 0 (0%) | 0 (0%) | 1 (100%) | 0 (0%) |
| Bacteria | Helicobacteracaeae | 1 | 0 (0%) | 0 (0%) | 1 (100%) | 0 (0%) |
| Bacteria | Lactobacillaceae | 1 | 1 (100%) | 0 (0%) | 1 (100%) | 0 (0%) |
| Bacteria | Leptospiraceae | 1 | 1 (100%) | 0 (0%) | 1 (100%) | 1 (100%) |
| Bacteria | Mycobacteriaceae | 2 | 0 (0%) | 0 (0%) | 2 (100%) | 0 (0%) |
| Bacteria | Mycoplasmataceae | 1 | 0 (0%) | 0 (0%) | 0 (0%) | 1 (100%) |
| Bacteria | Nocardiaceae | 1 | 0 (0%) | 0 (0%) | 1 (100%) | 1 (100%) |
| Bacteria | Pasteurellaceae | 3 | 3 (100%) | 3 (100%) | 0 (0%) | 0 (0%) |
| Bacteria | Staphylococcaceae | 1 | 1 (100%) | 1 (100%) | 0 (0%) | 0 (0%) |
| Bacteria | Streptococcaceae | 2 | 2 (100%) | 1 (50%) | 0 (0%) | 1 (50%) |
| Bacteria | Veillonellaceae | 1 | 1 (100%) | 1 (100%) | 0 (0%) | 0 (0%) |
| Fungi | Clavicipitaceae | 1 | 0 (0%) | 0 (0%) | 0 (0%) | 1 (100%) |
| Fungi | Enterocytozoonidae | 1 | 0 (0%) | 0 (0%) | 1 (100%) | 0 (0%) |
| Helminths | Trichinellidae | 1 | 1 (100%) | 0 (0%) | 1 (100%) | 0 (0%) |
| Protozoa | Cryptosporididae | 1 | 0 (0%) | 0 (0%) | 1 (100%) | 0 (0%) |
| Protozoa | Trypanosomatidae | 2 | 0 (0%) | 0 (0%) | 1 (50%) | 0 (0%) |
| Virus (DNA) | Adenoviridae | 1 | 1 (100%) | 1 (100%) | 0 (0%) | 1 (100%) |
| Virus (DNA) | Anelloviridae | 3 | 3 (100%) | 3 (100%) | 0 (0%) | 0 (0%) |
| Virus (DNA) | Circoviridae | 1 | 1 (100%) | 1 (100%) | 0 (0%) | 1 (100%) |
| Virus (DNA) | Hepadnaviridae | 1 | 1 (100%) | 1 (100%) | 0 (0%) | 0 (0%) |
| Virus (DNA) | Herpesviridae | 3 | 3 (100%) | 3 (100%) | 0 (0%) | 0 (0%) |
| Virus (DNA) | Parvoviridae | 8 | 8 (100%) | 8 (100%) | 0 (0%) | 0 (0%) |
| Virus (RNA) | Arteriviridae | 1 | 1 (100%) | 1 (100%) | 0 (0%) | 1 (100%) |
| Virus (RNA) | Bornaviridae | 1 | 0 (0%) | 0 (0%) | 0 (0%) | 0 (0%) |
| Virus (RNA) | Bunyaviridae | 2 | 0 (0%) | 0 (0%) | 2 (100%) | 0 (0%) |
| Virus (RNA) | Caliciviridae | 2 | 2 (100%) | 2 (100%) | 0 (0%) | 0 (0%) |
| Virus (RNA) | Coronaviridae | 2 | 1 (50%) | 1 (50%) | 1 (50%) | 0 (0%) |
| Virus (RNA) | Filoviridae | 1 | 0 (0%) | 0 (0%) | 1 (100%) | 0 (0%) |
| Virus (RNA) | Flaviviridae | 1 | 1 (100%) | 1 (100%) | 0 (0%) | 1 (100%) |
| Virus (RNA) | Hepeviridae | 1 | 0 (0%) | 0 (0%) | 1 (100%) | 0 (0%) |
| Virus (RNA) | Paramyxoviridae | 3 | 2 (67%) | 0 (0%) | 2 (67%) | 2 (67%) |
| Virus (RNA) | Picobirnaviridae | 1 | 1 (100%) | 1 (100%) | 0 (0%) | 0 (0%) |
| Virus (RNA) | Picornaviridae | 1 | 1 (100%) | 1 (100%) | 0 (0%) | 0 (0%) |
| Virus (RNA) | Reoviridae | 1 | 1 (100%) | 0 (0%) | 1 (100%) | 0 (0%) |
| Virus (RNA) | Rhabdoviridae | 1 | 0 (0%) | 0 (0%) | 1 (100%) | 0 (0%) |
