# Supplementary material for: Spatiotemporal trends in the discovery of new swine infectious agents
Source: Vet Res. 2015 Sep 28;46:114. doi: 10.1186/s13567-015-0226-8 (PMC4584486; doi:10.1186/s13567-015-0226-8)
Supplement: Additional file 4: — Infectious agent discoveries per country. An underlined country name means that this country is among the top 20% pig meat producing countries for the period 1985–2010. The variation in pig meat production from 1985 to 2010 was computed for the countries listed among the 50 largest pig meat producers in 1985. Countries for which this variation was higher than 50% are indicated by an asterisk. [file 13567_2015_226_MOESM4_ESM.docx]

| Countries | New species (%) | New variants (%) | Average pig meat  production (in tonnes) | Variation in  production (in %) |
| --- | --- | --- | --- | --- |
| China * | 8 (11%) | 13 (8%) | 31,911,380 | 194% |
| Germany | 8 (11%) | 10 (6%) | 4,299,692 | 19% |
| Japan | 7 (10%) | 13 (8%) | 1,362,386 | -16% |
| Canada * | 6 (8%) | 33 (19%) | 1,492,875 | 77% |
| UK | 6 (8%) | 11 (6%) | 901,956 | -22% |
| Australia | 6 (8%) | 8 (5%) | 343,995 | 29% |
| USA * | 4 (5%) | 13 (8%) | 8,364,629 | 52% |
| Spain * | 4 (5%) | 6 (3%) | 2,510,920 | 143% |
| Sweden | 3 (4%) | 3 (2%) | 292,350 | -21% |
| Portugal * | 2 (3%) | 4 (2%) | 298,845 | 120% |
| India | 2 (3%) | 3 (2%) | 420,672 | -8% |
| Switzerland | 2 (3%) | 2 (1%) | 247,994 | -12% |
| Brazil * | 2 (3%) | 2 (1%) | 2,082,654 | 310% |
| Belgium * | 2 (3%) | 2 (1%) | 969,554 | 57% |
| Denmark * | 1 (1%) | 18 (10%) | 1,512,924 | 54% |
| Hungary | 1 (1%) | 4 (2%) | 680,647 | -55% |
| Slovenia | 1 (1%) | 3 (2%) | 56,972 | - |
| Mexico | 1 (1%) | 2 (1%) | 987,835 | -9% |
| Ireland * | 1 (1%) | 2 (1%) | 198,556 | 59% |
| PNG | 1 (1%) | 1 (1%) | 53,923 | - |
| Philippines * | 1 (1%) | 1 (1%) | 1,082,817 | 310% |
| Norway | 1 (1%) | 1 (1%) | 102,073 | - |
| Nepal | 1 (1%) | 1 (1%) | 12,650 | - |
| Malaysia * | 1 (1%) | 1 (1%) | 213,758 | 53% |
| Cameroon | 1 (1%) | 1 (1%) | 18,950 | - |
| Venezuela | 0 (0%) | 3 (2%) | 132,334 | 43% |
| Slovakia | 0 (0%) | 2 (1%) | 196,434 | -64% |
| Netherlands | 0 (0%) | 2 (1%) | 1,485,551 | -9% |
| Korea * | 0 (0%) | 2 (1%) | 810,423 | 156% |
| Italy | 0 (0%) | 2 (1%) | 1,431,762 | 41% |
| Viet Nam * | 0 (0%) | 1 (1%) | 1,453,403 | 442% |
| Thailand * | 0 (0%) | 1 (1%) | 599,572 | 128% |
| Taipei | 0 (0%) | 1 (1%) | 970,786 | 2% |
| France | 0 (0%) | 1 (1%) | 2,085,882 | 36% |
